# Research evidence on the management of the cognitive impairment component of the post-COVID condition: a qualitative systematic review

**DOI:** 10.1192/j.eurpsy.2024.1770

**Published:** 2024-09-27

**Authors:** Antonio Melillo, Andrea Perrottelli, Edoardo Caporusso, Andrea Coltorti, Giulia Maria Giordano, Luigi Giuliani, Pasquale Pezzella, Paola Bucci, Armida Mucci, Silvana Galderisi, Mario Maj

**Affiliations:** 1 World Health Organization (WHO) Collaborating Center, University of Campania “Luigi Vanvitelli”, Naples, Italy; 2 Department of Biomedical and Dental Sciences and Morphofunctional Imaging, University of Messina, Messina, Italy

**Keywords:** cognitive impairment, COVID-19, long COVID, rehabilitation, treatment

## Abstract

**Background:**

Cognitive impairment (CI) is one of the most prevalent and burdensome consequences of COVID-19 infection, which can persist up to months or even years after remission of the infection. Current guidelines on post-COVID CI are based on available knowledge on treatments used for improving CI in other conditions. The current review aims to provide an updated overview of the existing evidence on the efficacy of treatments for post-COVID CI.

**Methods:**

A systematic literature search was conducted for studies published up to December 2023 using three databases (PubMed–Scopus–ProQuest). Controlled and noncontrolled trials, cohort studies, case series, and reports testing interventions on subjects with CI following COVID-19 infection were included.

**Results:**

After screening 7790 articles, 29 studies were included. Multidisciplinary approaches, particularly those combining cognitive remediation interventions, physical exercise, and dietary and sleep support, may improve CI and address the different needs of individuals with post-COVID-19 condition. Cognitive remediation interventions can provide a safe, cost-effective option and may be tailored to deficits in specific cognitive domains. Noninvasive brain stimulation techniques and hyperbaric oxygen therapy showed mixed and preliminary results. Evidence for other interventions, including pharmacological ones, remains sparse. Challenges in interpreting existing evidence include heterogeneity in study designs, assessment tools, and recruitment criteria; lack of long-term follow-up; and under-characterization of samples in relation to confounding factors.

**Conclusions:**

Further research, grounded on shared definitions of the post-COVID condition and on the accurate assessment of COVID-related CI, in well-defined study samples and with longer follow-ups, is crucial to address this significant unmet need.

## Introduction

The global confirmed case count of Coronavirus disease 2019 (COVID-19), caused by the severe acute respiratory syndrome coronavirus 2 (SARS-CoV-2), surpassed 775 million as of December 2023 [1]. COVID-19 is now considered a multisystemic condition, which can lead to a broad spectrum of symptoms and long-term sequelae that may persist after remission of the infection up to one year and – in a proportion of cases – is still present even after two years [2–9]. Different terms and definitions have been proposed for this persisting condition, such as long-Covid or post-COVID-19 condition (PCC), which was described by a Delphi consensus as a condition occurring in subjects with a history of SARS-CoV-2 infection, usually 3 months from the infection, with symptoms that last for at least 2 months [10]. Long-term sequelae include hyposmia/anosmia, fatigue, dyspnea, and neuropsychiatric conditions, such as depression, anxiety, and cognitive impairment (CI) [11, 12]. Among these, CI has been reported as one of the most prevalent and burdensome consequences of the infection, affecting over 20% of patients who contracted COVID-19 [4].

Although deficits in different cognitive domains have been described, those more frequently reported involve executive functions, speed of processing, attention, and working memory, which may lead to substantial detriments to the quality of life and daily functioning of individuals [13–15].

Despite the clinical relevance of post-COVID CI, several research questions remain open. First, its underlying pathophysiology is still unclear; several hypotheses have been proposed, including the direct infiltration of the virus in the central nervous system cells, or an indirect brain damage due to different mechanisms such as abnormal immunological response, inflammation, vasculopathy, or hypoxemia caused by the infection [16, 17]. Second, given the novelty of this condition, its natural course and long-term outcome are still unknown; according to recent evidence, post-COVID CI can show different trajectories, probably related to different pathogenesis: some individuals may experience early short-term CI that resolves within weeks or months, while in others it may persist even for two years [4, 6, 18–20]; in other cases, CI may emerge long after the acute infection has remitted, since the risk of developing post-COVID CI has been found still present even after 2 years from the acute infection [21]. Third, unlike other common symptoms of PCC, such as dyspnea and depression, there are no established and effective treatments for post-COVID CI [22].

The latest WHO recommendations [23] suggest the combination of multiple cognitive rehabilitation strategies including both restorative (e.g., repeated standardized cognitive exercises) and compensatory interventions (e.g., skills training on self-management strategies such as planning and prioritizing activities or simplifying large tasks into smaller components). However, the WHO guidelines identified no randomized or non-randomized controlled trials specifically testing the rehabilitation interventions for post-COVID CI; therefore, they were based on the evidence available for diverse populations, such as individuals with CI following traumatic brain injury and stroke-related CI [24]. Although findings collected in other populations affected by CI may still suggest valuable treatment strategies and clinical directions, there is a critical need for evidence-based therapeutic options specifically tailored to post-COVID CI. In fact, it is crucial to take into account the complexity of this condition, wherein patients may have CIs in one or more cognitive domains with a wide range of severity and duration [25]. Furthermore, comorbidity with somatic or psychiatric conditions in patients affected by post-COVID CI may exacerbate these deficits, potentially influencing the extent and nature of the CI and the efficacy of treatments. For instance, some studies addressing post-COVID CI showed that patients with PCC are often easily fatigued, which might affect a patient’s tolerance to the cognitive rehabilitation training [26].

Previous reviews that focused on treatment strategies for PCC, even when including the management of CI, did not provide a comprehensive overview on the topic due to the following limitations: in some cases, they addressed the efficacy of a single type of intervention (e.g., noninvasive brain stimulation [NIBS] [27]), retrieved studies in which cognitive deficits were not assessed [28, 29], or included only study protocols that have not provided yet results on the efficacy of treatments [30, 31]. Two recent qualitative reviews that specifically addressed the current research evidence on management of post-COVID CI [22, 32] highlighted the scarcity of data, as they only retrieved, respectively, three and four clinical trials specifically carried out in subjects with post-COVID CI. The authors concluded that different treatment options, including lifestyle interventions (e.g., sleep management, physical activity, and dietary interventions), cognitive training programs, and possibly anti-dementia drugs, should further be considered and tested. However, it should be noticed that these reviews did not report a detailed characterization of the studies in terms of inclusion and exclusion criteria, clinical and demographical variables of the samples, and employed cognitive tests. In addition, they also included studies in which differences in cognitive functioning before and after treatments were not assessed through objective measures [33, 34]; this is a crucial aspect given the discrepancy between subjective and objective cognitive assessments reported in patients with PCC [14].

In light of these observations and of the existing gaps in the current literature, the aims of the present systematic review are 1) providing an updated overview of the existing evidence on the efficacy of treatments implemented to improve cognitive functioning, assessed with objective assessments, in individuals suffering from post-COVID CI; 2) identifying potential limitations of the current evidence and providing methodological recommendations for further studies on the topic.

## Methods

The current systematic review was performed in line with the Preferred Reporting Items for Systematic Reviews and Meta-Analyses (PRISMA) guidelines [35]. A systematic literature search was conducted for studies published up to December 31, 2023, using three databases (PubMed, Scopus, and ProQuest). The following combination of search terms was used: (COVID-19 OR SARS-COV-2 OR 2019-nCoV OR “long covid” OR “persistent covid” OR “post covid” OR “long-haul covid” OR “Post-covid brain fog”) AND (cognition OR neurocognition OR “cognitive deficit” OR “cognitive impairment”) AND (neuromodulation OR intervention OR training OR stimulation OR remediation OR management OR treatment OR therap* OR rehabilitation). Duplicates from the combination of the three databases were excluded. Three investigators independently screened all articles for eligibility based on titles and abstracts, and then the full text of the selected articles was reviewed. Discrepancies in the selection of suitable articles were discussed by all authors and were resolved through discussion and consensus.

Inclusion criteria for the articles were established prior to the article review based on PICOS framework (Table 1).

**Table 1.** Inclusion criteria based on PICOS framework

Population (P):	Individuals reporting persisting symptoms following resolution of acute COVID–19 infection
Intervention (I):	Both pharmacological and non–pharmacological interventions related to the treatment of post–COVID–19 cognitive impairment
Comparison (C):	Pre−post–intervention comparison; if available, data on comparisons with treatment–as–usual or placebo groups will be reported
Outcome (O):	Cognitive functioning pertaining to at least one cognitive domain assessed by means of standardized tests, test batteries, or structured or semi–structured interviews
Study design (S):	Controlled and noncontrolled trials, cohort studies, case series, and reports

Exclusion criteria were 1) articles published before the pandemic; 2) studies considering non-interventional trials, or trials on interventions aimed at preventing post-COVID CI; 3) studies addressing not relevant populations (e.g., individuals with no history of acute infection or with a diagnosis of cognitive deficit preceding the COVID-19 infection); 4) studies testing the efficacy of treatments based only on self-report measures, given the evidence that these assessments are not as reliable and standardized as objective measures [23]; 5) preclinical and nonhuman studies; and 6) articles with unavailable full text in English.

No article was excluded based on study design, sample size, demographic characteristics of the subjects, applied definition or duration of the PCC, or time elapsed since the acute infection.

For the articles meeting inclusion criteria, data extraction included authors; year of publication; design of the study; sample size; demographic and clinical characteristics of the patients (including age, gender, years of education, duration of acute illness, duration of the PCC, history of hospitalization, or intensive care unit management); inclusion criteria; applied definition of PCC; tests used for assessing cognitive functioning; type, duration, and description of the employed intervention; assessment of confounding factors such as depressive and anxiety symptoms; statistical analysis; and main findings in relation to cognitive outcomes.

The methodological quality of the included studies was assessed using the Joana Briggs Institute Critical Appraisal tools [36].

## Results

### Search results

**Figure 1.**
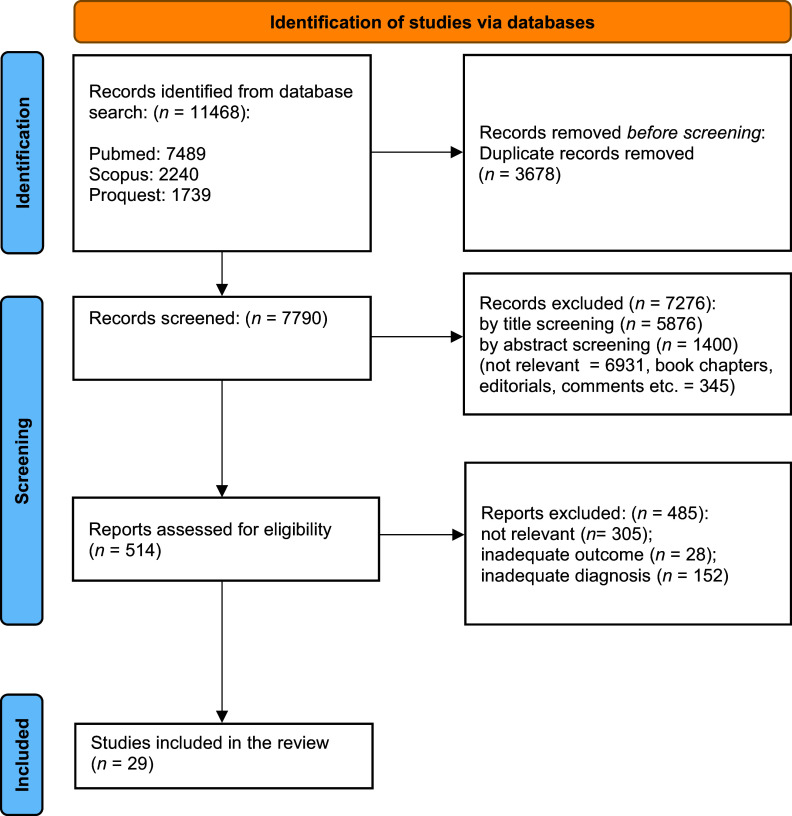
PRISMA flow diagram.

The combined outcome of the three database results included 11468 records (PubMed: 7489; Scopus: 2240; ProQuest: 1739). Details on screening, eligibility assessment, and exclusion criteria are reported in Figure 1.

A total of 29 articles met our criteria and were included in the review. Nine studies were randomized controlled trials (RCTs) [37–45], two non-RCTs [46, 47], nine noncontrolled trials [48–56], and nine were case reports [57–61] or case series [62–65].

In relation to the type of tested interventions, our search retrieved two articles on cognitive rehabilitation [46, 49], six on NIBS (one on transcranial alternating current stimulation [tACS] [59], two on transcranial direct current stimulation [tDCS] [41, 62], and three on transcranial magnetic stimulation [TMS] [48, 61, 64]), seven on pharmacological or nutraceutical compounds [40, 42, 43, 45, 50, 60, 63], three on hyperbaric oxygen therapy [HBOT] [37, 58, 65], one on neurofeedback training (NFT) intervention [57], one on photobiomodulation [38], one on meditation [39], and eight on multidisciplinary rehabilitation interventions [44, 47, 51–56].

### Inclusion criteria and clinical and demographic features of the enrolled subjects

Inclusion/exclusion criteria, when reported (22 studies), were heterogeneous with respect to the clinical condition required to be included. In most studies, the requirement for inclusion was the presence of self-reported post-COVID CI or other post-COVID symptoms; only in four studies, inclusion criteria listed the presence of post-COVID CI assessed by means of objective tests; in other four studies, only a history of hospitalization due to COVID-19 infection was listed as an inclusion criterion. Also with respect to the minimal duration of the PCC, inclusion criteria were quite heterogeneous (in the majority of studies 12 weeks, in others a duration ranging from 1 week to 6 months, or no temporal criterion at all). A detailed description of inclusion/exclusion criteria for all included studies is provided in the Supplementary Material (results section, paragraph 1, and Supplementary Table S1).

Demographic and clinical features of the subjects included in all studies are reported in Table 2 and Supplementary Table S1.

**Table 2.** Demographic and clinical features of retrieved samples

Sample size	**Intervention groups:** Mean: 24 SD = 21.78 Range: 1–73 Total samples: Mean: 33.1 SD = 26.99 Range: 1–100
Mean age across studies (min.–max.)	30 [61]–68 [50] years
Gender ratio (% of male subjects) across studies (min.–max.)	5% [49] and 66% [48] of males
Mean years of education across studies (min.–max.)	13.5 [40]–16.52 [41] years^a^
Mean duration of the PCC (min.–max.)	15 [42] and 340 days [64]^b^
Number of studies including individuals with history of hospitalization	16 [37, 41, 42, 46–50, 52–54, 56–58, 60, 64]^c^^,^^d^

a Data reported in 4 studies only [37, 40, 41, 62].

b Data missing in 12 studies [37–39, 43–46, 49, 50, 53, 54, 65].

c Seven studies included exclusively subjects with a history of hospitalization [42, 46, 47, 50, 56, 58, 60].

d Information was missing in 9 studies [38, 39, 43–45, 49, 54, 62, 65].

### CI assessment methodologies

High heterogeneity was observed among studies with regard to the tests and batteries employed for cognitive assessment. Details on the applied assessment tools in all included studies are reported in Table 3.

**Table 3.** Cognitive impairment assessment methodologies

Screening tools	MoCA: 7 studies [44, 45, 47, 51, 55, 56, 63] MMSE: 2 studies [50, 54] MoCA and MMSE: 2 studies [42, 60] BACS: 1 study [46]
Combination of screening batteries and individual tests	4 studies [38, 40, 60, 62]
Individual tests	4 studies TMT–A/B: 1 study [64] CR, PC: 1 study [39] TAP: 1 study [52] Stroop test: 1 study [41]
Comprehensive neuropsychological batteries	Mindstreams Computerized Cognitive Battery: 4 studies [37, 57, 58, 65] WMS–R: 2 studies [43, 61] WAIS–III: 1 study [53] WAIS–IV: 1 study [48] CAB: 1 study [49]

Abbreviations: BACS, Brief Assessment of Cognition in Schizophrenia scale; CAB, Cognitive Assessment Battery; CR, choice reaction time; MMSE, Mini Mental State Examination; MoCa, Montreal Cognitive Assessment; PC, Pattern Comparison Task; TAP, Test of Attentional Performance; TMT-A/B = Trail Making Test A and B; WAIS-IV, Wechsler Adult Intelligence Scale-IV; WMS-R: Wechsler Memory Scale-Revised.

### Methodological quality of the included studies

Applying the Critical Appraisal tools of the Joana Briggs Institute [36], fourteen of the included studies were categorized as being of good methodological quality [37, 41, 42, 45–50, 52, 57, 60, 61, 64], thirteen as being of average methodological quality [38, 40, 43, 44, 51, 53–56, 58, 59, 63, 65], and two as being of poor methodological quality [39, 62]. The main factors affecting the methodological quality were the poor characterization of experimental samples, the inadequate sensitivity of assessment tools, and the lack of control for confounding variables. Data on the risk of bias are reported in the Supplementary Material file (results section, paragraph 2).

### Results on the efficacy of the interventions

#### Cognitive remediation interventions

Cognitive remediation (CR) is defined as a behavioral training intervention targeting deficits in attention, memory, executive function, social cognition, or metacognition, using scientific principles of learning to improve cognitive skills and functional outcomes [66].

A non-RCT [46] tested in 15 patients the efficacy of the CogPack CR program consisting of 6 weekly sessions and tailored to the patients’ cognitive profiles, as assessed through the Brief Assessment of Cognition in Schizophrenia (BACS) scale. A significant improvement in global cognitive functioning was observed in the group of patients with respect to the control group [46]. Depressive symptomatology did not affect the efficacy of the CR intervention on global cognitive improvement [46].

A noncontrolled feasibility pilot study [49], testing an 8-week program of digital cognitive training, reported significant improvement in attention, memory, coordination, perception, and reasoning in a sample of 73 post-COVID individuals.

#### Noninvasive brain stimulation

NIBS refers to a range of techniques aimed at modulating brain electrical activity in targeted cortical areas, stimulating neuronal excitability, neural plasticity, and changes in connectivity patterns [67–69]. In the last decades, a range of different NIBS techniques, such as tACS, TMS, and tDCS, have been tested for the treatment of CI in different psychiatric and neurological conditions, as well as in healthy cognitive aging [70–78].

Three studies tested the efficacy of TMS [48, 61, 64]. The first was a pilot case series study in which 20 TMS sessions of intermittent theta-burst stimulation (iTBS) applied to the left dorsolateral prefrontal cortex (DLPFC) and to the right lateral orbitofrontal cortex (LOFC) led to an improvement in executive functions in a sample of 23 individuals [64]. A second noncontrolled study reported a significant post-intervention improvement in overall cognitive performance in 12 subjects receiving 10 TMS sessions applied to the frontal and occipital cerebral regions [48]. This study also reported that subjects showed an increase in blood flow in the frontal and occipital cortical areas, as compared to the pre-intervention assessment. A case report testing the effects of continuous accelerated theta-burst TMS applied to the right DLPFC, followed by intermittent accelerated theta-burst TMS applied to the left DLPFC, showed significant improvements in memory in a 30-year-old woman [61].

The efficacy of tDCS was tested in two studies. One randomized sham-controlled trial reported no significant effects on a task assessing executive functioning and processing speed in 23 subjects receiving eight tDCS sessions [41]. A case series on patients receiving tDCS combined with online cognitive training showed improvement in processing speed, verbal learning, and memory in the four included subjects [62].

As to tACS, one case report showed that 13 tACS sessions led to an improvement in attention, executive functions, verbal learning, and verbal memory, but not in working memory in a 40-year-old woman [59].

#### Pharmacological and nutraceutical interventions

Seven trials on potential pharmacological intervention for post-COVID CI were completed, while others, mainly testing the efficacy of anti-dementia drugs [22, 79], are currently ongoing.

One RCT [43] tested the efficacy of donepezil chlorhydrate, a cholinesterase inhibitor which has shown neuroprotective and anti-inflammatory effects and is approved for the treatment of Alzheimer’s disease [80, 81]. The study reported no differences between the intervention group (N = 15) and the control group (N = 10) on the overall score in memory performance, assessed through a test battery, after 4 and 12 weeks of treatment [43].

A second RCT tested the efficacy of the coordination complex between ethylmethylhydroxypyridine and trimethylhydrosinium propionate with succinate acid anion (CCSA), a newly marketed compound with potential neuroprotective effects, in 15 subjects as compared to 15 controls receiving placebo, and reported a significant improvement in cognitive performance only in the intervention group [45].

The efficacy of famotidine, a selective histamine H2 receptor antagonist, was tested in a RCT versus placebo, reporting that this drug significantly improved global cognition in the treatment group (N = 25), with no significant correlation with the improvements in depression and anxiety symptoms [42].

Another RCT tested the efficacy versus placebo of ultramicronized palmitoylethanolamide (PEA), an endocannabinoid drug which has been tested in several neurological and neurodegenerative conditions for its modulating role in neuroinflammation and synaptic neurotransmission [82, 83] combined with luteolin. The analysis showed no significant improvements in global cognitive functioning in both the PEA and control groups (*n* = 34) [40].

A noncontrolled study investigating the effects of a nutraceutical including different compounds such as L-theanine, vitamin B6, vitamin D, biotin, folic acid, and vitamin B12 found an improvement of the global MoCA scores, particularly within the attention and executive functioning domains in a sample of 40 elderly patients [50].

A case series study testing the benefits of *Ginkgo biloba* extract EGb 761 reported improvements in global cognitive functioning in five patients [63].

Finally, a case report of a patient found an improvement in global cognitive functioning and more specifically in the executive functions and verbal fluency domains, following the administration of perispinal etanercept, a tumor necrosis factor inhibitor [60].

#### Hyperbaric oxygen therapy

HBOT, the therapeutic administration of 100% oxygen at environmental pressures greater than one atmosphere, has been recently tested off-label for the treatment of CI associated with neurological disorders [84, 85], particularly in the case of traumatic brain injury [86] and vascular dementia [87].

In relation to post-COVID CI, a randomized sham-controlled trial tested the efficacy of HBOT [37] reporting significant improvements in global cognitive functioning, attention, and executive functions after HBOT in 37 subjects, as compared to the control group. Furthermore, the recovery of these cognitive domains was explained by neuroimaging data that suggested a restoration of functional connectivity between the frontoparietal, default mode and salience networks after the intervention [88].

A case series reported that global cognition, executive functions, attention, processing speed, and verbal fluency improved in a sample of 10 patients following 10 sessions of HBOT [65].

Furthermore, a case report on a single patient showed that 20 sessions of HBOT significantly improved both pulmonary capacity and global cognitive functioning [58]. The improvements were associated with an increase in brain perfusion assessed through MRI [58].

#### Neurofeedback training

NFT is an electroencephalogram-based biofeedback technique aimed at training self-regulation of neurophysiological states to reach specifically targeted electroencephalography (EEG) signals [89, 90].

One case report showed the effects of combining EEG-based NFT and goal-oriented cognitive training in the treatment of post-COVID CI [57]. The study involved a Sensory-Motor Rhythm and theta/beta training interventions showing improvements in attention, visual learning, memory, and executive functioning after 30 sessions during a period of 15 weeks [57].

#### Other interventions

A pilot study compared the efficacy of either transcranial or whole-body photo-biomodulation (PBM), a technique implementing ultraviolet rays with anti-inflammatory properties, reporting that both interventions were associated with significant improvement in cognitive performance in a sample of 14 individuals [38].

Furthermore, a RCT tested the efficacy of a meditation program and found an improvement in processing speed in a sample of 17 individuals [39].

#### Multidisciplinary interventions

Among studies included in this review, the highest number (N = 8) tested the efficacy of multidisciplinary interventions on CI. Of these, six [44, 51–54, 56] involved cognitive rehabilitation (five with restorative interventions [44, 52–54, 56] and one with compensatory interventions [51]).

A non-RCT testing the efficacy of in-person and supervised physical exercise (PE) together with dietary modules reported that the intervention group (N = 21) significantly improved in global cognitive functioning, as compared to the usual care group (N = 23) [47]. However, an RCT testing a remote-based digital multidisciplinary intervention including physical and cognitive exercise modules, as well as dietary and sleep hygiene recommendations, found no significant differences in cognitive performance improvements between subjects assigned to the multidisciplinary intervention (N = 52) and the control group (N = 48) [44].

The remaining six studies were all noncontrolled.

One study investigated the efficacy of a multidisciplinary intervention that included individual and group-based cognitive behavioral therapy (CBT) [91–95], individual and group-based cognitive training, and PE training during a 5-week stay in a rehabilitation facility in 80 subjects [52]. No significant improvements in memory and attention performance were observed at discharge [52].

Another study investigated the efficacy of an 8-week multidisciplinary intervention comprising both physical rehabilitation and digital CR interventions and reported improvements in verbal fluency, verbal learning, and memory, in a sample of 40 individuals [53].

The efficacy of a 30-day-long multidisciplinary intervention comprising both CR and physiotherapy was tested in a study that found significant improvements in global cognitive functioning, particularly in the domains of attention, abstract reasoning, memory, and visuospatial orientation in a sample of 64 individuals [56].

A cohort study on the efficacy of aerobic exercise, combined with educational sessions on fatigue, memory and concentration, and sleeping hygiene, reported a significant improvement in global cognitive functioning in a sample of 32 subjects [51].

A study testing the efficacy of a rehabilitation intervention consisting of physical and cognitive training reported improvements in attention, calculation, memory, and global functioning in a sample of 42 elderly men (65–80 years) recovering from hospitalization due to COVID-19 infection [54].

Finally, a study testing a 3-month multidisciplinary intervention including physical training and psychological, dietary, and occupational assistance reported an improvement in global functioning scores in a sample of 22 subjects [55].

## Discussion

Due to the dearth of evidence on the treatment of post-COVID CI, current guidelines are mainly based on the management of CI in other conditions [23, 25]. The present review retrieved 29 studies testing the efficacy of a broad range of interventions for post-COVID CI.

Evidence in six studies [47, 51, 53–56] included in the present review, of either good [47] or average [51, 53–56] methodological quality and carried out in relatively large samples [47, 53, 54, 56], shows that multidisciplinary interventions encompassing different components, such as physical rehabilitation interventions, cognitive training, and support for lifestyle modifications (e.g., dietary recommendations and sleep hygiene), can significantly improve post-COVID CI. It could be hypothesized that multidisciplinary approaches target the different components of PCC, which, particularly in some cases (subjects experiencing also sleep disturbances, mood and anxiety symptoms, and physical fatigue), may impact cognitive performance, as well as overall functional outcomes and quality of life. However, two studies testing multidisciplinary interventions reported no significant benefits [44, 52]. In one of them [44], which tested an app-based multidisciplinary intervention, the results might have been hindered by very low user compliance [44]. The advantages of app- and home-based multidisciplinary interventions are the low costs and their easy dissemination; however, their acceptability and feasibility need to be investigated in further trials. In the second study [52], a 5-week multidisciplinary intervention in a rehabilitation clinic setting did not significantly improve attention and working memory, despite remission of depressive symptomatology. However, this study did not assess other cognitive domains or global functioning at the post-intervention visit, which hinders comparison with other studies [52]. Consistently with this finding, another study [43] found no significant improvement in either attention or working memory, evaluated with specific individual tests. Therefore, further studies should investigate the effectiveness of multidisciplinary interventions using comprehensive cognitive batteries.

It is worth noticing that all the multidisciplinary interventions included PE [44, 51–56], which is considered a cornerstone of the rehabilitation from COVID-19 infection; in one study [51], the sole combination of aerobic exercise and education on compensatory CR strategies (e.g., pacing strategies and management of daily activities) was found to improve global cognitive performance [51]. Besides targeting physical fatigue, physical activity may improve cognition through its effects on hormonal and cardiovascular systems, as well as through its modulatory effects on neuroplasticity and inflammatory cascades [96].

Six of the studies on multidisciplinary interventions involved cognitive rehabilitation programs, either restorative – in the majority of cases – or compensatory [44, 51–54, 56]. In addition, the application of cognitive training interventions alone was also reported to effectively improve cognitive functioning in two other studies [46, 49]. Digital [53, 97] or virtual reality [98, 99] CR interventions can be home based and self-administered [49], thus providing a cost-effective intervention, which can be tailored to target specific cognitive domains based on patients’ characteristics [100–103]. Further RCT trials are needed in order to gain stronger evidence on the efficacy of these interventions and identify the most effective programs (e.g., CR exercises [44, 46, 49, 52–54, 56] or compensatory strategies [51]) and the best methods of administration (e.g., home-based [49] or supervised face-to-face interventions [46]).

Overall, our results also support the WHO recommendations on the management of post-COVID CI, which advise the use of a combination of restorative and compensatory cognitive rehabilitation interventions; the retrieved evidence, however, additionally suggests that these interventions may be more effective in the context of multidisciplinary approaches, when complemented by interventions such as PE, targeting the different symptoms that individuals may experience and that may contribute to cognitive and functional impairment.

In relation to NIBS techniques, their effects on neuronal responsiveness, long-term potentiation, and neurovascular modulation might improve the cortical hypometabolism described in the PCC [27, 59]; intermittent theta-burst TMS stimulation, through its facilitatory role on the DLPFC and by enhancing theta-gamma coupling involved in cognitive functions, has been suggested to improve cognitive functioning and stimulate neuroplasticity [64]. The retrieved evidence, however, was limited to one pilot study, one case series, and case reports for TMS [48, 61, 64], while only one case series and a single sham-controlled RCT were available for tDCS [41, 62], and the latter did not find positive effects on cognition [41]. This lack of efficacy might be explained by the fact that NIBS interventions have been reported to improve only specific cognitive domains, particularly working memory in the case of TMS, [70] and attention/vigilance and working memory for tDCS [72, 73]. Therefore, further studies using more comprehensive test batteries are needed to investigate their efficacy and whether specific post-COVID cognitive phenotypes can benefit from the use of these techniques.

Two case studies and one randomized sham-controlled trial reported evidence in favor of the efficacy of HBOT. Given its potential mechanisms on mitochondrial activity, neurogenesis, and angiogenesis [58], HBOT could be useful particularly in the case of patients who had suffered from hypoxia during acute COVID-19 illness and required treatment with high-flow oxygen [58]. In the retrieved sham-controlled trial [37], HBOT was associated with functional connectivity and white matter modifications, and the authors hypothesized that structural and functional connectivity analysis may represent both a treatment eligibility and response monitoring tool [88].

Evidence was sparse and limited to single studies for other interventions, including the pharmacological ones [40, 42, 43, 45, 50, 60, 63, 104]. Given the increased risk of developing dementia associated with the PCC [105, 106] and the similarities between post-COVID CI and dementia [22], the early start of anti-dementia drugs could be hypothesized for subsets of patients at a particularly higher risk of CI related to PCC, such as older subjects and those who experienced greater COVID-19 severity symptoms. However, the only retrieved trial testing the efficacy of an anti-dementia drug in individuals with PCC did not support its application [43].

Overall, the evidence collected up to this date by the current review is quite hard to interpret and summarize due to several methodological factors. First, the included studies showed heterogeneity in relation to inclusion and exclusion criteria, particularly regarding the applied definition of PCC and the recruitment time range from acute illness. Such heterogeneity, which was found to be diffuse in the current literature [22, 107–109], is particularly crucial, given the current uncertainty over the longitudinal trajectory of post-COVID-19 CI and consequentially the identification of the optimal time window for treatment [10, 25, 110].

Furthermore, the applied assessment tools for CI were also variable, with most of the studies including only screening tools, such as MoCA or MMSE, which might have inadequate sensitivity to assess improvements in cognitive functioning, as compared to neuropsychological test batteries that provide in-depth characterization of cognitive domains [50, 111]. Another factor to be taken into account is that many of the included studies recruited subjects based on self-reported CI, but employed objective assessment tools for the pre–post evaluation of treatment efficacy [112–115]. However, a meta-analysis focusing on COVID-19 patients showed that studies applying objective assessment tests reported significantly greater rates of individuals with CI in comparison with those employing self-reporting tools (36% versus 18%, respectively), which might suggest that the population who might benefit from treatments to improve CI might be much larger than the number of individuals with subjective complaints and may thus be not adequately represented in samples recruited through self-reported CI as the main inclusion criterion [14].

In addition, all studies lacked a long-term longitudinal design to analyze the stability over time of the reported improvements [111]. Furthermore, the absence of control groups in many studies, together with the long duration of the intervention protocols, does not allow to control for placebo effects and for the potential spontaneous remission of this condition, respectively. Finally, experimental samples were heterogeneous and under-characterized in relation to several parameters related to potential confounding factors, such as the symptom severity of the acute infection, the occurrence of hospitalization, and the presence of comorbidities during the PCC, such as psychiatric conditions [116–119].

With the end of the pandemic outbreak of COVID-19 and the emergence of new and milder variants of the disease, individuals are facing lower risks of both severe acute manifestations and sequelae; however, evidence shows that new variants of the COVID-19 virus are currently associated with similar risk of CI and overall neurological and psychiatric sequalae as compared to earlier variants [21]. This evidence indicates that health services worldwide will continue to face high rates of post-COVID CI and PCC diagnoses [120] and, together with the evidence described in the present paper, strongly suggests that further research is needed to address this largely unmet need. Future research will need to be grounded on well-established definitions of PCC and post-COVID CI, assess objectively CI through comprehensive cognitive batteries, and employ longitudinal evaluations and study designs that allow better stratification of the studied population and control for confounding factors.

## Supporting information

Melillo et al. supplementary material Melillo et al. supplementary material

## Supplementary material

The supplementary material for this article can be found at http://doi.org/10.1192/j.eurpsy.2024.1770.
