# Supplementary material for: Research evidence on the management of the cognitive impairment component of the post-COVID condition: a qualitative systematic review
Source: Eur Psychiatry. 2024 Sep 27;67(1):e60. doi: 10.1192/j.eurpsy.2024.1770 (PMC11457117; doi:10.1192/j.eurpsy.2024.1770)
Supplement: Melillo et al. supplementary material [file S092493382401770Xsup001.docx]

**Supplementary material:**

Research evidence on the management of the cognitive impairment component of the post-COVID condition: a qualitative systematic review

**Results:**

1. Inclusion criteria of included studies

2. Risk of bias assessment

**Tables:**

Table S1. Description of the studies included: demographic and clinical characteristics of the samples and inclusion criteria of the studies

Table S2. Characteristics and outcomes of the interventions

**Results**

**1. Inclusion criteria of included studies**

Thirteen studies enrolled subjects on the basis of self-reported post-COVID-19 symptoms [1-13]. Out of these, 5 studies enrolled subjects with any post-covid symptom [5, 7, 9-11], 3 enrolled subjects exclusively on the basis of cognitive complaints [1, 2, 4], 3 enrolled subjects with post-covid fatigue (either physical or cognitive) [3, 6, 8], 1 study enrolled subjects with either depressive, anxiety symptoms, self-reported CI or sleeping problems [12], 1 study with either self-reported depressive symptoms or cognitive impairment [13]. Only 4 studies enrolled subjects on the basis of the presence of CI assessed through objective tests [14-17]. Finally, 1 study included subjects who were referred to rehabilitation services through a discharge follow up pathway at COVID-19 medical follow up or from their GP [18], and 4 studies enrolled subjects on the basis of hospitalization during the acute phase of the infection [19-22]. Inclusion/exclusion criteria were not applicable for the remaining studies [23-29].

In relation to the required minimum duration of the post-COVID-19 condition (PCC), 9 studies required subjects to have symptoms persisting at least 12 weeks from the acute phase of the infection [1, 3-5, 7-11], while other studies employed different criteria: 1 study enrolled subjects with symptoms persisting at least five months after infection [2], 2 studies enrolled subjects one month after discharge [14, 22], one study seven days after acute symptom elapse [16], 1 study only established a maximum time distance from acute infection of six months [17], 8 studies did not establish any temporal criterion [6, 12, 13, 15, 18-21]. For a detailed description of the inclusion criteria and of the duration of the PCC, please refer to **Table S1**.

**2. Risk of Bias assessment**

Among the retrieved studies, 14 were categorized as being of good methodological quality [1, 3, 4, 8-10, 13, 14, 16, 17, 19, 23, 24, 29], 13 as being of average methodological quality [2, 5-7, 11, 15, 18, 20-22, 25, 27, 28] and 2 as being of poor methodological quality [12, 26].
The main factors affecting the methodological quality of the retrieved studies was the characterization of studies samples. In fact, only few studies characterized in detail their study samples, reporting data on the level of education [1, 6, 10, 26], the rates of hospitalization [2, 4, 5, 7, 8, 12, 15, 26] and admission to intensive care units [1, 2, 5, 7, 8, 12, 15, 26] and the duration of PCC or the time elapsed between the study recruitment and the acute infection [1, 2, 4, 5, 7, 8, 12, 14, 15, 17] of the subjects included. Additionally, the studies included often employed assessment tools with inadequate sensitivity [2, 7, 8, 16-21, 28, 29]. Finally, most studies, except 3 [1, 9, 14], did not check for the effects of potential confounding factors on CI, such as the presence of comorbid psychiatric symptoms.

**Tables**

**Table S1. Description of the included studies: demographic and clinical characteristics of the samples and inclusion criteria of the studies**

| **First Author,**  **year, study design** | **Intervention type** | **Sample size / Demographic characteristics of included subjects** | **Duration of COVID-19 acute infection and post-COVID condition and frequency of patients hospitalized** | **Inclusion/Exclusion criteria of the study** |
| --- | --- | --- | --- | --- |
| Duñabeitia et al. 2023  [4] **Non-controlled trial** | **CRT** | Total Subjects (n): 73 Mean Age (years): 46.1  Gender (n. of males): 7 (9.5%) Level of education (years): N/A | DAI (mean days): N/A  DOI (mean days): N/A  Subjects with history of hospitalization in acute phase (n): N/A  Subjects with history of ICU management (n): N/A | Self-reported Post-covid cognitive impairment for more than three months. |
| Palladini et al. 2022  [14] **Non-Randomized controlled trial** | **CRT** | Total Subjects (n): 45  Intervention group (n): 15  Mean Age (years): 56.80  Gender (n. of males): 9 (60%) Level of education (years): N/A  Control group (n): 30  Mean Age (years): 59.60  Gender (n. of males): 23 (77%) Level of education (years): N/A | Intervention group:  DAI (mean days): N/A  DOI (mean days): N/A  Subjects with history of hospitalization in acute phase (n): 15 (100%)  Subjects with history of ICU management (n): 0%  Control group: DAI (mean days): N/A  DOI (mean days): N/A  Subjects with history of hospitalization in acute phase (n): 30 (100%)  Subjects with history of ICU management (n): 0% | Individuals with cognitive impairment as assessed through objective screening test (BACS), one month after hospital discharge. |
| Sabel et al. 2021 [27] **Case report** | **tACS** | Total Subjects (n): 1 Age (years): 40  Gender: female Level of education (years): N/A | DAI (mean days): 14  DOI (days): 270  Subjects with history of hospitalization in acute phase (n): 0 (0%)  Subjects with history of ICU management (n): 0 (0%) | Inclusion/exclusion criteria not applicable. Patient reported cognitive impairment and blurred vision persisting post-acute illness. |
| Cavendish et al., 2022 [26] **Case series** | **tDCS** | Total Subjects (n): 4  Mean Age (years): 50.5  Gender (n. of males): 1 (25%)  Level of education (years): 14 | DAI (mean days): N/A  DOI (mean days): 90  Subjects with history of hospitalization in acute phase (n): N/A  Subjects with history of ICU management (n): N/A | Inclusion/exclusion criteria not applicable. Patient reported fatigue, cognitive impairment and emotional symptoms persisting post-acute illness |
| Oliver-Mas et al. 2023 [10] **Double-blind, parallel-group, sham-controlled pilot study** | **tDCS** | Total Subjects (n): 46  Intervention group (n): 23 Mean Age (years): 47.26  Gender (n. of males): 8 (34.79%) Level of education (years): 16.52  Sham group (n): 23 Mean Age (years): 44.11  Gender (n. of males): 2 (8.34%) Level of education (years): 15.91 | Intervention group: DAI (mean days): N/A  DOI (mean days): 559.5  Subjects with history of hospitalization in acute phase (n): 4 (17.4%)  Subjects with history of ICU management (n):1 (4.3%)  Sham group: DAI (mean days): N/A  DOI (mean days): 678.2  Subjects with history of hospitalization in acute phase (n): 3 (12.5%)  Subjects with history of ICU management (n): 1 (4.2%) | Self-reported PCC at least 3 months after infection with symptoms lasting for at least 2 months. |
| Noda et al. 2022 [13] **Case series** | **TMS** | Total Subjects (n): 23 Mean Age (years): 38.2  Gender (n. of males): 10 (43.5%) Level of education (years): N/A | DAI (mean days): N/A  DOI (mean days): 340.2 days  Subjects with history of hospitalization in acute phase (n): 7 (30%)  Subjects with history of ICU management (n): 0 (0%) | Self-reported cognitive impairment or depressive symptoms. |
| Sasaki et al., 2023 [3] **Non-controlled trial** | **TMS** | Total Subjects (n): 12 Mean Age (years): 44.3  Gender (n. of males: 8 (66.7%) Level of education (years): N/A | DAI (mean days): N/A DOI (mean days): 202.4  Subjects with history of hospitalization in acute phase (n): 2 (20%)  Subjects with history of ICU management (n): 0 (0%) | Self-reported physical and cognitive fatigue for at least 3 months following COVID-19 infection. |
| Chang et al. 2023 [24] **Case report** | **TMS** | Total Subjects (n): 1 Age (years): 30  Gender: female  Level of education (years): N/A | DAI (mean days): 7 DOI (mean days): 60  Subjects with history of hospitalization in acute phase (n): 0 (0%)  Subjects with history of ICU management (n): 0 (0%) | Inclusion/exclusion criteria not applicable. Patient reported anxiety, depression, insomnia, and brain fog persisting for 8 weeks post-acute illness. |
| Bove et al. 2021 [17] **Non-controlled trial** | **Pharmacological/nutraceutical intervention: Synaid** | Total Subjects (n): 40  Hospitalized group (n): 19 (47.5%) Mean Age (years): 68  Gender (n. of males): N/A  Level of education (years): N/A  Home-cared group (n): 21 (52,5%)  Gender (n. of males): N/A  Level of education (mean years): N/A | Hospitalized group: DAI (mean days): N/A  DOI (mean days): N/A Subjects with history of hospitalization in acute phase (n): 19 (100%)  Subjects with history of ICU management (n): 0 (0%)  Home-cared group: DAI (mean days): N/A  DOI (mean days): N/A Subjects with history of hospitalization in acute phase (n): 0 (0%)  Subjects with history of ICU management (n): 0 (0%) | Individuals with MMSE scores between 20 and 27 and with subjective cognitive impairment recovered from COVID-19 during the last 6 months. |
| Momtazmanesh et al. 2023 [16] **Randomized, placebo-controlled, double-blind trial** | **Pharmacological/nutraceutical intervention: Famotidine** | Total Subjects (n): 50  Intervention group (n): 25  Mean Age (years): 37.32  Gender (n. of males): 14 (56.0%) Level of education (years): N/A  Placebo group (n): 25  Mean Age (years): 35.16  Gender (n. of males): 13 (54.0%) Level of education (years): N/A | Intervention group: DAI (mean days): N/A  DOI (mean days): 15  Subjects with history of hospitalization in acute phase (n): 25 (100%)  Subjects with history of ICU management (n): 2 (8%)  Placebo group: DAI (mean days): N/A  DOI (mean days): 15  Subjects with history of hospitalization in acute phase (n): 25 (100%)  Subjects with history of ICU management (n): 2 (8%) | Patients with a history of hospitalization, recruited at least 7 days after elapse of acute symptoms and with a score lower than 22 for the MMSE or 23 for the MoCA. |
| Pooladgar et al. 2023 [15] **Randomized, single-blind, parallel controlled trial** | **Pharmacological/nutraceutical intervention: Donepezil hydrochloride** | Total Subjects (n): 25  Intervention group (n): 10  Mean Age (years): 46  Gender (n. of males): 5 (50%) Level of education (years): N/A  Placebo group (n): 15  Mean Age (years): 43.87  Gender (n. of males): 6 (40%) Level of education (years): N/A | Intervention group: DAI (mean days): N/A  DOI (mean days): N/A  Subjects with history of hospitalization in acute phase (n): N/A  Subjects with history of ICU management: N/A  Control group: DAI (mean days): N/A  DOI (mean days): N/A  Subjects with history of hospitalization in acute phase (n): N/A  Subjects with history of ICU management (n): N/A | Self-reported complaint of memory impairment and deviation in memory scores (less than 85) on at least one index of the WMS-R. |
| Tanashyan et al. 2023 [8] **Randomized double-blind placebo- controlled trial** | **Pharmacological/nutraceutical intervention: CCSA treatment** | Total Subjects (n): 30  Intervention group (n): 15  Mean Age (years): 42.0  Gender (n. of males): 2 (13%) Level of education (years): N/A  Placebo group (n): 15 subjects Mean Age (years): 28  Gender (n. of males): 3 (27%) Level of education (years): N/A | Intervention group: DAI (mean days): N/A  DOI (mean days): N/A  Subjects with history of hospitalization in acute phase (n): N/A  Subjects with history of ICU management (n): N/A  Placebo group: DAI (mean days): N/A  DOI (mean days): N/A  Subjects with history of hospitalization in acute phase (n): N/A  Subjects with history of ICU management (n): N/A | Self-reported physical and mental fatigue (MFI-20 scale) between 3 and 12 months after acute COVID-19. |
| Tobinick et al. 2022 [29] **Case report** | **Pharmacological/nutraceutical intervention:**  **Perispinal Etanercept (PSE)** | Total Subjects (n): 1 Age (years): 48  Gender: female Level of education (years): N/A | DAI (mean days): 14  DOI (mean days): 365  Subjects with history of hospitalization in acute phase (n): 1 (100%)  Subjects with history of ICU management (n): 0 (0%) | Inclusion/exclusion criteria not applicable. Patient reported fatigue and cognitive impairment persisting post-acute illness. |
| Versace et al., 2022 [6] **Double-blind, placebo-controlled, randomized clinical trial** | **Pharmacological/nutraceutical intervention:**  **co-ultramicronized palmitoylethanolamide/luteolin (PEA-LUT)** | Total Subjects (n): 34  Intervention group (n): 17 Mean Age (years): 53.5  Gender (n. of males): 6 (35.29%) Level of education (years): 13.7  Placebo group (n): 17 Mean Age (years): 48.1  Gender (n. of males): 6 (35.29%) Level of education (years): 13.5 | Intervention group:  DAI (mean days): N/A  DOI (mean days): 291.4  Subjects with history of hospitalization in acute phase (n): 0  Subjects with history of ICU management (n): 0  Placebo group: DAI (mean days): N/A  DOI (mean days): 290.2 (97.7)  Subjects with history of hospitalization in acute phase (n): 0  Subjects with history of ICU management (n):0 | Self-reported cognitive or physical fatigue following mild COVID-19 acute infection. |
| Zifko et al. 2022 [28] **Case series** | **Pharmacological/nutraceutical intervention:**  **EGb 761** | Total Subjects (n): 5  Mean Age (years): 34.6  Gender (n. of males): 2 (40%)  Level of education (years): N/A | DAI (days): N/A  DOI (mean days): 135.8  Subjects with history of hospitalization in acute phase (n): 0 (0%)  Subjects with history of ICU management (n): 0 | Inclusion/exclusion criteria not applicable. Patient reported fatigue and cognitive impairment persisting post-acute illness |
| Bhaiyat et al. 2022  [25] **Case report** | **HBOT** | Total Subjects (n): 1  Age (years): 55  Gender: male  Level of education (years): N/A | DAI (days): 26 DOI (days): 90  Subjects with history of hospitalization in acute phase (n): 1 (100%)  Subjects with history of ICU management (n): 0 | Inclusion/exclusion criteria not applicable. Patient reported fatigue and cognitive impairment persisting post-acute illness |
| Robbins et al. 2021 [5] **Case series** | **HBOT** | Total Subjects (n): 10 Mean Age (years): 47.5  Gender (n. of males): 4 (40%) Level of education (years): N/A | DAI (mean days): N/A  DOI (mean days): N/A  Subjects with history of hospitalization in acute phase (n): N/A  Subjects with history of ICU management (n): N/A | Self-reported PCC for more than 12 weeks. |
| Zilberman-Itskovich et al. 2022 [1] **Randomized, sham-controlled, double blind trial** | **HBOT** | Total Subjects (n): 73  HBOT group (n): 37  Mean Age (years): 48.4  Gender (n. of males): 18 (48%)  Level of education (years): 14.6  Control group (n): 36  Mean Age (years): 47.8  Gender (n. of males): 11 (30.6%)  Level of education (years): 15.1 | HBOT group: DAI (mean days): N/A  DOI (mean days): 159.1 Subjects with history of hospitalization in acute phase (n): 4 (10.8%)  Subjects with history of ICU management (n): N/A  Control group: DAI (mean days): N/A DOI (mean days): 171.5 days  Subjects with history of hospitalization in acute phase (n): 8 (22%)  Subjects with history of ICU management (n): N/A | Self-reported PCC for at least 3 months. |
| Łuckoś et al. 2021 [23] **Case report** | **Neurofeedback plus Goal-oriented Cognitive Training** | Total Subjects (n): 1  Age (years): 49  Gender: female  Level of education (years): N/A | DAI (days): N/A DOI (days): 150 days  Subjects with history of hospitalization in acute phase (n): 0 (0%)  Subjects with history of ICU management (n): 0 | Inclusion/exclusion criteria not applicable. Patient reported cognitive impairment persisting post-acute illness |
| Bowen et al. 2022 [2] **Randomized head-to-head trial** | **Photobiomodulation** | Total Subjects (n): 14  Mean Age (years): 56  Gender (n. of males): 4 (28.5%)  Level of education (years): N/A | DAI (mean days): N/A DOI (mean days): N/A  Subjects with history of hospitalization in acute phase (n): N/A  Subjects with history of ICU management (n): N/A | Self-reported PCC for at least 5 months. |
| Hausswirth et al. 2023 [12] **Randomized controlled trial** | **Neuro-Meditation Program**  **on Cognitive Function** | Total Subjects (n): 49  Intervention group (n): 17  Mean Age (years): 47.1  Gender (n. of males): 4 (23%) Level of education (years): N/A  Control group (n): 17  Mean Age (years): 48.7  Gender (n. of males): 5 (29%) Level of education (years): N/A  Healthy group (n): 15  Mean Age (years): 45.9  Gender (n. of males): 5 (33.3%) Level of education (years): N/A | Intervention group:  DAI (mean days): N/A  DOI (mean days): N/A  Subjects with history of hospitalization in acute phase (n): N/A Subjects with history of ICU management (n): 2 (12%)  Control group: DAI (mean days): N/A  DOI (mean days): N/A  Subjects with history of hospitalization in acute phase (n): N/A  Subjects with history of ICU management (n): 1 (6%) | Subjects with history of COVID-19 infection and scores of HADS-Anxiety >8 and/or HADS-Depression >8 and/or, CFQ >4 and/or SSQ >17. |
| Albu et al. 2021  [11]  **Non-controlled trial** | **Multidisciplinary intervention** | Total Subjects (n): 40 Mean Age (years): 52  Gender (n. of males): 24 (60%) Level of education (years): N/A | DAI (mean days): 56.6  DOI (mean days): 90  Subjects with history of hospitalization in acute phase (n): 30 (75%)  Subjects with history of ICU management (n):21 (53%) | Individuals with neurological, cognitive  and musculoskeletal sequelae and persistent symptoms of COVID-19 infection (more than 3 months after initial symptoms). |
| Amini et al. 2021  [20]  **Non-controlled trial** | **Multidisciplinary intervention** | Total Subjects (n): 42  Mean Age (years): 70.03  Gender (n. of males): 42 (100%)  Level of education (years): N/A | DAI (days): N/A DOI (days): N/A  Subjects with history of hospitalization in acute phase (n): 0 (100%)  Subjects with history of ICU management (n): 0 | Elder adults recovering from COVID-19 infection recently discharged from hospital. |
| Asimakos et al. 2023 [19] **Non-randomized parallel assignment two-arm trial** | **Multidisciplinary intervention** | Total Subjects (n): 52  Intervention group (n): 21  Mean Age (years): 55  Gender (n. of males): 9 (47%) Level of education (years): N/A  Usual care group (n): 23  Mean Age (years): 60  Gender (n. of males): 11 (48%) Level of education (years): N/A | Intervention group: DAI (mean days): 25  DOI (mean days): 78  Subjects with history of hospitalization in acute phase (n):100%  Subjects with history of ICU management (n): 17  Usual care group: DAI (mean days): N/A  DOI (mean days): 72  Subjects with history of hospitalization in acute phase (n): 100%  Subjects with history of ICU management (n): 15 | Patients who were hospitalised in a medical ward or in ICU requiring high oxygen mixtures, non-invasive ventilation (NIV) or intubation. |
| Daynes et al. 2021 [18] **Non-controlled trial** | **Multidisciplinary intervention** | Total Subjects (n): 30 Mean Age (years): 58  Gender (n. of males): 16 (52%) Level of education (years): N/A | DAI (mean days): N/A  DOI (mean days): 125.5  Subjects with history of hospitalization in acute phase (n): 26 (87%)  Subjects with history of ICU management (n): 5 (16.7%) | Individuals with rehabilitative needs either referred through a discharge follow up pathway at COVID-19 medical follow up or referral from their general practitioners. |
| Everaerts et al. 2021  [21]  **Non-controlled trial** | **Multidisciplinary intervention** | Total Subjects (n): 22 Mean Age (years): 54.5  Gender (n. of males): 15 (68%) Level of education (years): N/A | DAI (mean days): 29  DOI (mean days): N/A  Subjects with history of hospitalization in acute phase (n): 22 (100%)  Subjects with history of ICU management (n): 15 (68%) | Patients who were hospitalised, in a medical ward or in ICU, requiring high oxygen mixtures, non-invasive ventilation (NIV) or intubation. |
| Kupferschmitt et al. 2023 [9] **Non-controlled trial** | **Multidisciplinary intervention** | Total Subjects (n): 80  Mean Age (years): 50.89  Gender (n. of males): 26 (32%) Level of education (years): N/A | DAI (mean days): N/A  DOI (mean days): N/A  Subjects with history of hospitalization in acute phase (n): 8 (22%)  Subjects with history of ICU management (n): 4 (5%) | Self-reported PCC for at least 12 weeks after infection. |
| Rabaiotti et al. 2023  [22]  **Non-controlled trial** | **Multidisciplinary intervention** | Total Subjects (n): 64 Mean Age (years): 67.3  Gender (n. of males: 42 (65.6%) Level of education (years): N/A | DAI (mean days): 55.8  DOI (mean days): 90  Subjects with history of hospitalization in acute phase (n): 64 (100%)  Subjects with history of ICU management (n): 40 (63%) | Patients admitted to the sub-intensive or intensive care units for COVID-19, and had developed post-covid symptoms with or without cognitive impairment. |
| Samper‑Pardo et al. 2023 [7] **Randomized controlled trial** | **Multidisciplinary intervention** | Total Subjects (n): 100  Intervention group (n): 52 subjects Mean Age (years): 48.25  Gender (n. of males): 8 (15.4%) Level of education (years): N/A  Control group (n): 48 subjects Mean Age (years): 48.3  Gender (n. of males): 12 (25%) Level of education (years): N/A | Intervention group: DAI (mean days): N/A  DOI (mean days): 472,5  Subjects with history of hospitalization in acute phase (n): N/A  Subjects with history of ICU management (n): N/A  Control group: DAI (mean days): N/A  DOI (mean days): 486  Subjects with history of hospitalization in acute phase (n): N/A  Subjects with history of ICU management (n): N/A | Self-reported PCC for at least 12 weeks after the acute infection. |

**BACS**= Brief Assessment of Cognition in Schizophrenia;**CBT**= Cognitive Behavioral Therapy; **CCT**= Personalized Computerized Cognitive Training; **CCSA**= coordination complex with succinate acid anion; **CI**= Cognitive impairment; **CMT=** Cognitive-Motor Training; **CRT**= Cognitive remediation therapy; **DAI**= Duration of acute COVID-19 illness; **DOI**= Duration of Post-Covid Condition Illness ; **EGb 761®**= Ginkgo biloba special extract; **HBOT**= hyperbaric oxygen therapy; **ICU**= Intensive Care Unit; **MADRS**= Montgomery–Åsberg Depression Rating Scale; **MFI-20**= Multidimensional Fatigue Inventory; **MMSE**= Mini-Mental State Examination; **MoCA**= Montreal Cognitive Assessment; **N/A**= Not Available; **PEA-LUT**= Palmitoylethanolamide and Luteoline; **PCC**= Post-Covid Condition;; **tDCS**= Transcranial Direct Current Stimulation; **tACS**= transcranial alternating current stimulation; **tPBM**= Transcranial photobiomodulation; **TMS**= Transcranial Magnetic Stimulation;; **wb-PBM**= whole body photobiomodulation; **WMS-R**= Wechsler Memory Scale-Revised.

**Table S2. Description of the included studies: characteristics and outcomes of the interventions**

| **First author,**  **year, study design** | **Details on the intervention employed** | **Cognitive domains assessed and tests used** | **Time of evaluation after treatment** | **Outcome of the intervention** | **Evaluation of psychiatric symptoms** |
| --- | --- | --- | --- | --- | --- |
| Duñabeitia et al. 2023 [4] **Non-controlled trial** | **CRT**  Daily home-based personalized computerized cognitive training (CCT) sessions during an 8-week period. | **Global Cognitive Functioning:** CAB™ PRO  **A/V:** CAB™ PRO  **PS:** CAB™ PRO  **EF:** CAB™ PRO | Immediately after the intervention (8 weeks) | Consistent increase in cognitive performance after CCT and particularly in attention, memory, coordination, perception, and reasoning. | N/A |
| Palladini et al. 2022 [14] **Non-Randomized controlled trial** | **CRT**  Two months face-to-face CRT program carried out through COGPACK software. | **Global Cognitive Functioning**: BACS  **A/V**: BACS  **PS**:BACS  **VF**: BACS  **EF**: BACS  **WM:** BACS | Immediately after the intervention (2 months) | Significant improvement in global cognition. Significant improvement in time among both treated and untreated patients in executive functions, and verbal fluency, while only the treated patients showed improvement in psychomotor coordination. | Depressive symptoms (ZSDS) both at baseline and follow-up. Using depressive symptoms as a covariate for the analysis did not affect efficacy of CRT on global cognitive improvement. |
| Sabel et al. 2021 [27] **Case report** | **tACS**  Average of 11 sessions.  <2 mAmp tACS plus psychological counselling and relaxation technique training. | **A/V**: Tap Test, Go- No Go test  **EF:** Tap test  **WM:** Tap test  **VELM:** RAVLT | 2 and 3 weeks after the intervention | Improvement in A/V, EF and VeLM domains, but not in WM. | N/A |
| Cavendish et al., 2022 [26] **Case series** | **tDCS  plus online cognitive training**  20 daily 20-min sessions. Bilateral prefrontal tDCS plus online cognitive training using the BrainHQ platform. | **Global Cognitive Functioning**: MoCA  **A/V**: TEADI, TEADCO  **PS**: FDT  **VeLM**: RAVLT  **ViLM**: ROCF | Immediately after the intervention (20 days) | Trends of improvement in PS and VeLM. | Depressive symptoms assessed at through QIDS; anxiety symptoms assessed through QIDS, PANAS+/- and STAI. Correlation between psychiatric symptoms and cognition was not investigated. |
| Oliver-Mas et al. 2023 [10] **Double-blind, parallel-group, sham-controlled pilot study** | **tDCS**  8 sessions during two consecutive weeks.  Anode was placed over the left DLPFC. The cathode was placed on the contralateral supraorbital region. | **PS, EF:** Stroop test | Immediately after the intervention (two weeks) and one month later | No significant improvement in PS and EF after completion of tDCS sessions. | Depressive symptoms (BDI) present in 20 participants (42.55%) and anxiety symptoms (STAI) in 30 subjects (63.82%) at baseline. Depressive symptoms improved both in the intervention and placebo groups, after study completion. |
| Noda et al. 2022 [13] **Case series** | **TMS**  20 sessions. SEMI-piTBS for the left DLPFC and low frequency repetitive TMS for the right LOFC. | **EF:** TMT-A, TMT-B | Immediately after the intervention (5 weeks) | Improvement in EF. | Depressive symptoms (HADS) scores improved significantly after intervention. Correlation between depression and cognitive functioning was not analyzed. |
| Sasaki et al., 2023 [3] **Non-controlled trial** | **TMS**  10 sessions once every 1–2 weeks.  10-sec stimulations  at 10 Hz and 10-sec intervals applied to the occipital region | **Global Cognitive Functioning:** WAIS-4 full-scale IQ index  **PS:** WAIS-4 processing speed index  **WM:**  WAIS-4 WM index  **VELM:** WAIS-4 verbal comprehension index  **ViLM=** WAIS- 4 Perceptual Reasoning Index | Immediately after the intervention (10 weeks) | Significant improvement in all the cognitive domains assessed after intervention. | N/A |
| Chang et al. 2023 [24] **Case report** | **TMS**  10 sessions twice daily over 5 days. Continuous accelerated Theta-Burst TMS applied to the right DLPFC, followed by intermittent Accelerated Theta-Burst TMS applied to the left DLPFC. | **Global Cognitive Functioning, PS, VF, WM:** WMS | Immediately after the intervention (5 days) | Improvement in WMS-Logical Memory, WMS-Faces, WMS-Verbal Paired Associates, and WMS-Family Pictures scores. | Depressive and anxiety symptoms improved after the intervention (BDI, HAM-D and BAI). |
| Bove et al. 2021 [17] **Non-controlled trial** | **Pharmacological/nutraceutical intervention:**  **Synaid**  1 capsule per day for 12 weeks | **Global Cognitive Functioning**: MMSE | Immediately after the intervention (3 months) | Slight, but statistically significant, improvement in global cognitive functioning in the treatment group. | Depressive symptoms (Self-Rating Depression Scale) improved both in the intervention and placebo groups. |
| Momtazmanesh et al. 2023 [16] **Randomized, placebo-controlled, double-blind trial** | **Pharmacological/nutraceutical intervention:**  **Famotidine**  80 mg per day for 12 weeks | **Global Cognitive Functioning:** MMSE, MoCA | Evaluated at baseline, at 6 week, and at 12 week from the start of the intervention | Higher MMSE and MoCA scores in the intervention group vs. the placebo group both at week 6 and 12, and as compared to baseline scores. | Intervention group experienced a larger reduction in depressive symptoms (HAM-D) as well as in anxiety scores (HAM-A), as compared to the placebo group. Correlation with cognitive functioning was not investigated. |
| Pooladgar et al. 2023 [15] **Randomized, single-blind, parallel controlled trial** | **Pharmacological/nutraceutical intervention:**  **Donepezil**  Donepezil 5 mg per day for 12 weeks | **Global Cognitive Functioning, A/V, PS, EF, VF, WM:** WMS-R | Evaluated at baseline, at 4 weeks, and at 12 weeks from the start of the intervention | Significant improvement only in the Visual Reproduction I and Verbal Paired Associates II subtests and only in the intervention group. | N/A |
| Tanashyan et al. 2023 [8] **Randomized double-blind placebo- controlled trial** | **Pharmacological/nutraceutical intervention:**  **CCSA treatment**  Intramuscular injection of 500 mg of ethylmethylhydroxypyridine succinate and 500 mg of meldonium (CCSA)/5 ml for 10 days. | **Global Cognitive Functioning:** MoCA | Immediately after the intervention (10 days) | Improvement in global functioning across time were significantly higher in the CCSA group, as compared to the placebo group. | N/A |
| Tobinick et al. 2022 [29] **Case report** | **Pharmacological/nutraceutical intervention:**  **Perispinal Etanercept (PSE)**  Single dose 25 mg perispinal etanercept | **Global Cognitive Functioning**: BDI-II, FAS, MMSE, MoCA  **PS**: TMT-A and TMT-B  **VF**: COWAT, MACE  **EF**: COWAT, TMT-A and -B  **VF:** MMSE, MoCA | Immediately after the intervention and after 29 days | Improvement in EF, VF and global cognitive functioning (both after the intervention and at 1-month follow-up visit). | Depressive symptoms (BDI-II) were reduced at both post-intervention evaluations, as compared to the baseline. |
| Versace et al., 2022 [6] **Double-blind, placebo-controlled, randomized clinical trial** | **Pharmacological/nutraceutical intervention:**  **co-ultramicronized palmitoylethanolamide/luteolin (PEA-LUT)**  PEA-LUT 700 mg + 70 mg or PLACEBO, administered orally for eight weeks | **Global Cognitive Functioning:**  MoCA  **EF:** Frontal Assessment Battery | Immediately after the intervention (3 months) | No significant improvement in cognitive functioning. | N/A |
| Zifko et al. 2022 [28] **Case series** | **Pharmacological/nutraceutical intervention:**  **Treatment with EGb 761**  2 x 80 mg EGb 761® daily | **Global Cognitive Functioning**: MoCA | Immediately after the intervention (11weeks on average) | Improvement in global cognitive functioning and in the A/V and EF domains. | N/A |
| Bhaiyat et al. 2022  [25] **Case report** | **HBOT**  60 HBOT sessions, 5 days per week. 100% oxygen at 2 ATA for minutes 90 | **Global Cognitive Functioning**: Mindstreams computerized cognitive battery  **A/V**: Go-No Go test, Choice reaction time test  **PS**: Staged Information Processing  **VF**: COWA, Boston Naming test, WAIS-III similarities subtest  **EF**: Clock Drawing Test, TMT-A, DSST WMS-III Mental Control subtest  **VeLM**: RAVLT, WMS-III Logical Memory subtest  **ViLM**: WMS-III Block Design subtest | Immediately after the intervention (3 months) | The patient showed improvement in all cognitive domain assessed. | N/A |
| Robbins et al. 2021 [5] **Case series** | **HBOT**  10 HBOT daily sessions over 12 days 100% oxygen at 2.4 atmospheres for 1 hour and 45 minutes. | **Global Cognitive Functioning, A/V, PS, EF,  VeLM,ViLM**: Mindstreams computerized cognitive battery | Evaluated 10 days after treatment initiation | Improvement in global cognitive functioning, EF, A/V, PS and VF. | N/A |
| Zilberman-Itskovich et al. 2022 [1] **Randomized, sham-controlled, double blind trial** | **HBOT**  40 HBOT daily sessions, five sessions per week within a two-month period.  100% oxygen by mask at 2 ATA for 90 min. | **Global Cognitive Functioning**: Mindstreams computerized cognitive battery  **A/V**: Go-No Go test, Choice reaction time test  **PS**: Staged Information Processing  **VF**: COWA, Boston Naming test, WAIS-III similarities subtest  **EF**: Clock Drawing Test, TMT-A, DSST WMS-III Mental Control subtest  **VeLM**: RAVLT, WMS-III Logical Memory subtest  **ViLM**: WMS-III Block Design subtest | Immediately after the intervention (2 months) | Improvement in global cognitive functioning, A/V and EF, but not in PS, VF, VeLM, ViLM. Improvement recorded in the HBOT group were significantly higher than in the control group. | Anxiety symptoms (BSI-18) significantly improved in the HBOT group across time, while they did not change in the control group. |
| Łuckoś et al. 2021 [23] **Case report** | **Neurofeedback plus Goal-oriented Cognitive Training**  Neurofeedback sessions and goal-oriented Cognitive Training twice a week for 15 weeks. | **Global Cognitive Functioning**: Mindstreams computerized cognitive battery  **A/V**: TMT-A and B  **PS**: Staged Information Processing  **VF**: Boston Naming test  **EF**: SCWT, TMT-A and B, DSST WCST  **VeLM**: RAVLT, WMS-III verbal Memory subtests, TT  **ViLM**: WMS-III Block Design subtest | Immediately after the intervention (15 weeks) | Improvement in global cognitive functioning, particularly in ViLM, A/V, EF. | N/A |
| Bowen et al. 2022 [2] **Randomized head-to-head trial** | **Photobiomodulation**  Three treatment sessions per week for 4 weeks.  One group of subjects completed the transcranial photobiomodulation, while the other group completed the whole-body photobiomodulation. | **Global Cognitive Functioning:** MoCA  **PS**: TMT-A, TMT-B, physical reaction time  **EF**: DSST | Immediately after the intervention (4 weeks) | For Group 1 (transcranial photobiomodulation), improvement for global cognitive functioning, EF and PS.   For Group 2 (Whole-body photobiomodulation), improvement in global cognitive functioning , EF while in PS, only TMT-A improved. | N/A |
| Hausswirth et al. 2023 [12] **Randomized controlled trial** | **Neuro-Meditation Program**  **on Cognitive Function**  Ten 30-min sessions of Neuro-Meditation (Rebalance®)  over a period of five weeks. | **PS**: CR  **ViLM**: PC | Immediately after the intervention (5 weeks) and 1 week after intervention | Improvement in PS and ViLM. | Included subjects showed clinical levels in HADS-Anxiety and HADS-Depression mean scores at baseline. Anxiety and depression scores improved significantly after the intervention. Correlation with cognitive scores was not explored. |
| Albu et al. 2021  [11]  **Non-controlled trial** | **Multidisciplinary Rehabilitation**  8 weeks multidisciplinary rehabilitation comprising physical respiratory and cognitive rehabilitation. Cognitive rehabilitation was conducted online using the Guttmann, NeuroPersonalTrainer. | **A/V:**WAIS-III  **VF:**F-A-S test  **VeLM:** RAVLT, WAIS-III  **WM:** WAIS-III | Immediately after the intervention (8 weeks) | Improvement in two of the cognitive domains evaluated. | N/A |
| Amini et al. 2021  [20]  **Non-controlled trial** | **Multidisciplinary intervention**  4-week twice a week cognitive and motor training program. | **Global Cognitive Functioning:** MMSE  **A/V:** MMSE  **EF:** MMSE | Evaluated 2 weeks after treatment and 3 months after the intervention | Improvement in global cognitive functioning, AV and EF functioning both at short-term follow-up and long-term evaluation. | Scores of depression and anxiety (GHQ-2) improved significantly both at the short-term follow-up and at the long-term evaluation. |
| Asimakos et al. 2023 [19] **Non-randomized parallel assignment two-arm trial** | **Multidisciplinary Rehabilitation**  Two sessions per week during a 8-weeks period comprising supervised exercise training, breathing control, dietary advice and psychological support. | **Global Cognitive Functioning:** MoCA | Immediately after the intervention (8 weeks) | Improvement in global functioning. | Improvement in anxiety symptoms (HADS-A), but not in depressive symptoms (HADS-D), after treatment. Correlation with cognitive functioning was not investigated. |
| Daynes et al. 2021 [18] **Non-controlled trial** | **Multidisciplinary intervention**  6 weeks of physical rehabilitation intervention and educational sessions including pacing strategies, memory and attention compensation strategies | **Global Cognitive Functioning:** MoCA | Immediately after the intervention (6 weeks) | Significant improvement in global cognitive functioning in the intervention group. | No significant improvement in anxiety (HADS) and depressive symptoms (HADS) after the intervention. |
| Everaerts et al. 2021  [21]  **Non-controlled trial** | **Multidisciplinary Rehabilitation**  Exercise sessions of 1.5 hours three times a week for three months and support by physiotherapists, a psychologist, a social worker, a dietitian and an occupational therapist. | **Global Cognitive Functioning:** MoCA | Evaluated at baseline and after three months | Improvement in global cognitive functioning in individuals with cognitive impairment (n=10) at hospital discharge. | At 3 months, anxiety symptoms (HADS-A) were present in more patients, as compared to the baseline. |
| Kupferschmitt et al. 2023 [9] **Non-controlled trial** | **Multidisciplinary rehabilitation** Individual and group psychotherapy (CBT) cognitive training in a group setting (2 × 50 min per week) and in an individual setting (as needed), individualised aerobic exercise training, body awareness training, breathing therapy, relaxation techniques and social counselling. | **A/V, WM:** TAP | Immediately after the intervention (five weeks) | No significant improvement in A/V and WM after rehabilitation. | Significant improvement in depressive symptoms (PHQ-9) after treatment.   Significant correlations were present at baseline between cognitive impairment and psychiatric symptoms.  Changes in depression severity did not contribute to variance in cognitive functioning scores between admission and discharge. |
| Rabaiotti et al. 2023  [22]  **Non-controlled trial** | **Multidisciplinary rehabilitation**  30 days-long program comprising physiotherapy and cognitive remediation intervention. | **Global Cognitive Functioning:** MoCA | Immediately after the intervention (4 weeks) | Improvement in global cognitive functioning. | No significant improvement was found for depressive (HADS-D) and anxiety (HADS-A) symptoms. |
| Samper‑Pardo et al. 2023 [7] **Randomized controlled trial** | **Multidisciplinary rehabilitation**  Telerehabilitation  intervention using ReCOVery  APP for three months. Intervention comprised dietary and exercise recommendations, respiratory rehabilitation, cognitive exercises. | **Global Cognitive Functioning:** MoCA | Evaluated after 6 weeks from the start of the intervention and upon its competition (12 weeks) | Improvement in cognitive functioning over time did not significantly differ between groups. | No significant improvement was found for depressive (HADS-D) and anxiety (HADS-A) symptoms in both groups. |

**ATA=** atmosphere absolute pressure; **A/V**= Attention/Vigilance; **BACS**= Brief Assessment of Cognition in Schizophrenia; **BAI**= Beck Anxiety Inventory; **BDI-II**= Beck Depression Inventory-II; **BSI-18**= Brief Symptom Inventory; **CAB**= Cognitive Assessment Battery; **CBT**= Cognitive Behavioral Therapy; **CCT**= Personalized Computerized Cognitive Training; **CCSA**= coordination complex with succinate acid anion; **CFQ***= Chalder Fatigue Scale; **CFQ**= Cognitive Failure Questionnaire; **CI**= Cognitive impairment; **CMT=** Cognitive-Motor Training; **COWAT**= Controlled Oral Word Association test; **CRT**= Cognitive remediation therapy; **CR**= Choice reaction time; **DAI**= Duration of acute COVID-19 illness; **DLPFC=** dorsolateral prefrontal cortex; **DOI**= Duration of Post-Covid Condition Illness; **DSST**= Digit Symbol Substitution test; **EF**= Executive Functions; **EGb 761®**= Ginkgo biloba special extract; **FAB**= Frontal Assessment Battery; **FAS**= Fatigue Assessment Scale; **FSS**= Fatigue Severity Scale; **FDT**= Five Digit Test; **GHQ-2**= General Health Questionnaire; **HADS**= Hospital Anxiety and Depression Scale; **HAM-A**= Hamilton-Anxiety scale; **HAM-D**= Hamilton-depression scale; **HBOT**= hyperbaric oxygen therapy; **HD-tDCS**= High-Definition transcranial Direct Current Stimulation; **ICU**= Intensive Care Unit; **LOFC**= lateral orbitofrontal cortex **MACE**= Military Acute Concussion Evaluation; **MADRS**= Montgomery–Åsberg Depression Rating Scale; **MFI-20**= Multidimensional Fatigue Inventory; **MMSE**= Mini-Mental State Examination; **MoCA**= Montreal Cognitive Assessment; **N/A**= Not Available; **PANAS+/-**= Positive (+) and Negative (-) Affect Scale; **PEA-LUT**= Palmitoylethanolamide and Luteoline; **PHQ-9**=Patient Health Questionnaire-9; **PS**= Processing Speed; **PSE**= Perispinal Etanercept; **PC**= Pattern Comparison Task; **PCC**= Post-Covid Condition; **POMS**= Profile of Mood States; **QIDS**= Quick Inventory of Depressive Symptomatology; **RAVLT**= Rey Auditory Verbal Learning Test; **ROCF**= Rey-Osterrieth Complex Figure e Recall; **SCWT**= Stroop Color And Word Test;; **SEMI-piTBS=** semi-prolonged intermittent theta burst stimulation; **SSQ**=Spiegel Sleep Quality questionnaire; **STAI**= The State-Trait Anxiety Inventory; **tDCS**= Transcranial Direct Current Stimulation; **TEADI**= Test of Divided Attention; **TACS**= transcranial alternating current stimulation; **TAP**= Test of Attentional Performance; **tPBM**= Transcranial photobiomodulation; **TEADCO**= Test of Concentrated Attention; **TMS**= Transcranial Magnetic Stimulation; **TMT-A**= Trail-making Test - A; **TMT-B**= Trail-making Test - B; **TT**= Token Test; **VeLM**= Verbal Learning and Memory; **VF**= Verbal Fluency; **ViLM**= Visual Learning and Memory; **WAIS**= Wechsler Adult Intelligence Scale; **wb-PBM**= whole body photobiomodulation; **WCST**= Wisconsin Card Sorting Test; **WMS-III**= Wechsler Memory Scale, 3rd Edition; **WMS-R**= Wechsler Memory Scale-Revised; **ZSDS**= Zung self-rating depression scale

**References**

[1] Zilberman-Itskovich S, Catalogna M, Sasson E, Elman-Shina K, Hadanny A, Lang E, et al. Hyperbaric oxygen therapy improves neurocognitive functions and symptoms of post-COVID condition: randomized controlled trial. Sci Rep. 2022;12(1):11252. https://doi.org/10.1038/s41598-022-15565-0.

[2] Bowen R, Arany PR. Use of either transcranial or whole-body photobiomodulation treatments improves COVID-19 brain fog. J Biophotonics. 2023:e202200391. https://doi.org/10.1002/jbio.202200391.

[3] Sasaki N, Yamatoku M, Tsuchida T, Sato H, Yamaguchi K. Effect of Repetitive Transcranial Magnetic Stimulation on Long Coronavirus Disease 2019 with Fatigue and Cognitive Dysfunction. Prog Rehabil Med. 2023;8:20230004. https://doi.org/10.2490/prm.20230004.

[4] Duñabeitia JA, Mera F, Baro Ó, Jadad-Garcia T, Jadad AR. Personalized Computerized Training for Cognitive Dysfunction after COVID-19: A Before-and-After Feasibility Pilot Study. Int J Environ Res Public Health. 2023;20(4):3100. https://doi.org/10.3390/ijerph20043100.

[5] Robbins T, Gonevski M, Clark C, Baitule S, Sharma K, Magar A, et al. Hyperbaric oxygen therapy for the treatment of long COVID: early evaluation of a highly promising intervention. Clin Med (Lond). 2021;21(6):e629-e32. https://doi.org/10.7861/clinmed.2021-0462.

[6] Versace V, Ortelli P, Dezi S, Ferrazzoli D, Alibardi A, Bonini I, et al. Co-ultramicronized palmitoylethanolamide/luteolin normalizes GABA(B)-ergic activity and cortical plasticity in long COVID-19 syndrome. Clin Neurophysiol. 2023;145:81-8. https://doi.org/10.1016/j.clinph.2022.10.017.

[7] Samper-Pardo M, León-Herrera S, Oliván-Blázquez B, Méndez-López F, Domínguez-García M, Sánchez-Recio R. Effectiveness of a telerehabilitation intervention using ReCOVery APP of long COVID patients: a randomized, 3-month follow-up clinical trial. Sci Rep. 2023;13(1):7943. https://doi.org/10.1038/s41598-023-35058-y.

[8] Tanashyan M, Morozova S, Raskurazhev A, Kuznetsova P. A prospective randomized, double-blind placebo-controlled study to evaluate the effectiveness of neuroprotective therapy using functional brain MRI in patients with post-covid chronic fatigue syndrome. Biomed Pharmacother. 2023;168:115723. https://doi.org/https://doi.org/10.1016/j.biopha.2023.115723.

[9] Kupferschmitt A, Jöbges M, Randerath J, Hinterberger T, Loew TH, Köllner V. Attention deficits and depressive symptoms improve differentially after rehabilitation of post-COVID condition – A prospective cohort study. J Psychosom Res. 2023;175:111540. https://doi.org/https://doi.org/10.1016/j.jpsychores.2023.111540.

[10] Oliver-Mas S, Delgado-Alonso C, Delgado-Álvarez A, Díez-Cirarda M, Cuevas C, Fernández-Romero L, et al. Transcranial direct current stimulation for post-COVID fatigue: a randomized, double-blind, controlled pilot study. Brain Commun. 2023;5(2):fcad117. https://doi.org/10.1093/braincomms/fcad117.

[11] Albu S, Rivas Zozaya N, Murillo N, García-Molina A, Figueroa Chacón CA, Kumru H. Multidisciplinary outpatient rehabilitation of physical and neurological sequelae and persistent symptoms of covid-19: a prospective, observational cohort study. Disabil Rehabil. 2022;44(22):6833-40. https://doi.org/10.1080/09638288.2021.1977398.

[12] Hausswirth C, Schmit C, Rougier Y, Coste A. Positive Impacts of a Four-Week Neuro-Meditation Program on Cognitive Function in Post-Acute Sequelae of COVID-19 Patients: A Randomized Controlled Trial. Int J Environ Res Public Health. 2023;20(2):1361. https://doi.org/10.3390/ijerph20021361.

[13] Noda Y, Sato A, Fujii K, Nagano Y, Iwasa M, Hirahata K, et al. A pilot study of the effect of transcranial magnetic stimulation treatment on cognitive dysfunction associated with post COVID-19 condition. Psychiatry Clin Neurosci. 2023;77(4):241-2. https://doi.org/10.1111/pcn.13527.

[14] Palladini M, Bravi B, Colombo F, Caselani E, Di Pasquasio C, D’Orsi G, et al. Cognitive remediation therapy for post-acute persistent cognitive deficits in COVID-19 survivors: A proof-of-concept study. Neuropsychol Rehabil. 2023;33(7):1207-24. https://doi.org/10.1080/09602011.2022.2075016.

[15] Pooladgar P, Sakhabakhsh M, Soleiman-Meigooni S, Taghva A, Nasiri M, Darazam IA. The effect of donepezil hydrochloride on post-COVID memory impairment: A randomized controlled trial. J Clin Neurosci. 2023;118:168-74. https://doi.org/10.1016/j.jocn.2023.09.005.

[16] Momtazmanesh S, Ansari S, Izadi Z, Shobeiri P, Vatankhah V, Seifi A, et al. Effect of famotidine on cognitive and behavioral dysfunctions induced in post-COVID-19 infection: A randomized, double-blind, and placebo-controlled study. J Psychosom Res. 2023;172:111389. https://doi.org/10.1016/j.jpsychores.2023.111389.

[17] Bove M, Fogacci F, Quattrocchi S, Veronesi M, Cicero AFG. Effect of Synaid on cognitive functions and mood in elderly subjects with self-perceived loss of memory after COVID-19 infection. Arch Med Sci. 2021;17(6):1797-9. https://doi.org/10.5114/aoms/141502.

[18] Daynes E, Gerlis C, Chaplin E, Gardiner N, Singh SJ. Early experiences of rehabilitation for individuals post-COVID to improve fatigue, breathlessness exercise capacity and cognition - A cohort study. Chron Respir Dis. 2021;18:14799731211015691. https://doi.org/10.1177/14799731211015691.

[19] Asimakos A, Spetsioti S, Mavronasou A, Gounopoulos P, Siousioura D, Dima E, et al. Additive benefit of rehabilitation on physical status, symptoms and mental health after hospitalisation for severe COVID-19 pneumonia. BMJ Open Respir Res. 2023;10(1):e001377. https://doi.org/10.1136/bmjresp-2022-001377.

[20] Amini A, Vaezmousavi M, Shirvani H. The effectiveness of cognitive-motor training on reconstructing cognitive health components in older male adults, recovered from the COVID-19. Neurol Sci. 2022;43(2):1395-403. https://doi.org/10.1007/s10072-021-05502-w.

[21] Everaerts S, Heyns A, Langer D, Beyens H, Hermans G, Troosters T, et al. COVID-19 recovery: benefits of multidisciplinary respiratory rehabilitation. BMJ Open Respir Res. 2021;8(1):e000837. https://doi.org/10.1136/bmjresp-2020-000837.

[22] Rabaiotti P, Ciracì C, Donelli D, Oggioni C, Rizzi B, Savi F, et al. Effects of Multidisciplinary Rehabilitation Enhanced with Neuropsychological Treatment on Post-Acute SARS-CoV-2 Cognitive Impairment (Brain Fog): An Observational Study. Brain Sci. 2023;13(5):791. https://doi.org/10.3390/brainsci13050791.

[23] Łuckoś M, Cielebąk K, Kamiński P. EEG neurofeedback in the treatment of cognitive dysfunctions after the infection of SARS-COV-2 and long COVID-19. Acta Neuropsychologica. 2021;19(3):361-72. https://doi.org/https://doi.org/10.5604/01.3001.0015.2464.

[24] Chang CH, Chen SJ, Chen YC, Tsai HC. A 30-Year-Old Woman with an 8-Week History of Anxiety, Depression, Insomnia, and Mild Cognitive Impairment Following COVID-19 Who Responded to Accelerated Bilateral Theta-Burst Transcranial Magnetic Stimulation Over the Prefrontal Cortex. Am J Case Rep. 2023;24:e938732. https://doi.org/10.12659/ajcr.938732.

[25] Bhaiyat AM, Sasson E, Wang Z, Khairy S, Ginzarly M, Qureshi U, et al. Hyperbaric oxygen treatment for long coronavirus disease-19: a case report. J Med Case Rep. 2022;16(1):80. https://doi.org/10.1186/s13256-022-03287-w.

[26] Cavendish BA, Lima A, Bertola L, Charvet L, Bikson M, Brunoni AR, et al. Combination of transcranial direct current stimulation with online cognitive training improves symptoms of Post-acute Sequelae of COVID-19: A case series. Brain Stimul. 2022;15(6):1375-7. https://doi.org/10.1016/j.brs.2022.09.008.

[27] Sabel BA, Zhou W, Huber F, Schmidt F, Sabel K, Gonschorek A, et al. Non-invasive brain microcurrent stimulation therapy of long-COVID-19 reduces vascular dysregulation and improves visual and cognitive impairment. Restor Neurol Neurosci. 2021;39(6):393-408. https://doi.org/10.3233/rnn-211249.

[28] Zifko UA, Yacob M, Braun BJ, Dietz GPH. Alleviation of Post-COVID-19 Cognitive Deficits by Treatment with EGb 761®: A Case Series. Am J Case Rep. 2022;23:e937094. https://doi.org/10.12659/ajcr.937094.

[29] Tobinick E, Spengler RN, Ignatowski TA, Wassel M, Laborde S. Rapid improvement in severe long COVID following perispinal etanercept. Curr Med Res Opin. 2022;38(12):2013-20. https://doi.org/10.1080/03007995.2022.2096351.
